# Case of a Foreign Body in the Air Passages Three Months

**Published:** 1859-03

**Authors:** Daniel Brainard

**Affiliations:** Surgeon of the U. S. Marine Hospital, Chicago, Etc.


THE CHICAGO

### MEDICAL JOURNAL.

VOL. II. MARCH, 1859. No. 3.

### ORIGINAL COMMUNICATIONS.

ARTICLE I.

### CASE OF A FOREIGN BODY IN THE AIR PASSAGES THREE MONTHS.

SUCCESSFUL OPERATION, WITH SUGGESTIONS ON THE BEST MEANS OF PREVENTING HEMORRHAGE DURING THE OPERATION, AND OBVIATING THE EFFECTS OF THE ENTRANCE OF COLD AIR AFTER TRACHEOTOMY.

BY DANIEL BRAINARD, M.D., SURGEON OF U. S. MARINE HOSPITAL, CHICAGO, ETC.

AIR PASSAGES.

*Jan. 7th*, 1859.—A child two years old was brought to me, with symptoms of a foreign body in the bronchia. The parents stated that, three months previously, the child was seen to put a piece of the shell of a hazelnut in its mouth, and was instantly seized with symptoms of suffocation, so severe as to give apprehension of a fatal issue. From these the child recovered, but has been subject, ever since, to severe paroxysms of coughing, has lost its appetite, and is emaciated.

At present it presents all the appearances of a child affected with whooping cough, except the peculiar sound of inspiration in that disease. On examination of the chest, the respiratory murmur on the right side is found feeble, and a *sibilant ronchus* is distinctly heard, especially during inspiration. A mucous rattle is also perceptible over the whole of the right side of the chest. There is no dullness on percussion, nor bronchial respiration. There is an offensive odor of the breath, so marked as to be compared only with that perceived in gangrene of the lungs.

Believing these symptoms sufficiently indicated the presence of a foreign body, I proceeded, Jan. 8th, with the assistance of Prof. Byford and Drs. Powell and Durham, to perform the operation of tracheotomy.

After opening the trachea, I passed repeatedly a curved probe downwards, and to the right side, to loosen the foreign body. This produced severe efforts at coughing, and I was about to relinquish all attempts to dislodge it, when about a teaspoonful of thick pus presented itself at the opening, and immediately after it the foreign body itself, which was easily removed.

The child was kept under the influence of chloroform during the whole of the operation; and there is none in which the anæsthetic is more useful.

After the operation, a full dose of black drop was administered. The child soon fell asleep, and continued to rest well during the night. At times, it drank milk freely.

*Jan. 9th.*—The child appears well in every respect, excepting that the foul odor of the breath continues. It breathes principally through the larynx, the piece of the trachea raised having been left to assume its natural position.

*Jan. 13th.*—Child improving; has occasional paroxysms of coughing. Takes its food; appears lively.

*Jan. 14th.*—The child has been affected with twitchings; febrile action. Respiration quickened.

*Jan. 19th.*—Appears well; playful; appetite good; respiration natural, little cough, and not much mucous in the air passages.

Returned home. Some days afterward I heard of its safe arrival and continued improvement, and have since learned the cure was perfect.

### SUGGESTION OF IMPROVEMENTS IN THE OPERATION.

The suggestions which I have to offer in regard to tracheotomy, relate to the means of preventing hemorrhage, to keeping the opening made by the operation pervious without resorting to a tube, and at the same time controlling the entrance of cold air abruptly into the lungs.

1. *Hemorrhage.*—No experienced surgeon will deny that this is a serious difficulty in this operation. M. Guersent, surgeon of the children's hospital at Paris, than whom there is no better authority on this subject, relates several cases where death occurred from opening large vessels, and one where it happened from the division of the thyroid veins.* Syncope is a frequent effect of this loss of blood, and even suffocation may occur. M. Guersent advises sucking the blood out of the trachea with a catheter, when this is threatened.

In order to prevent hemorrhage, I proceed in the following manner: Having incised the skin and facia by successive and careful incisions, I press the sterno hyoid and sterno thyroid muscles to each side with the fingers, and thus expose the thyroid body. This effected, I pass under the isthmus a director curved, or an aneurismal needle. This is followed by a common suture needle, which may be passed with the blunt end foremost, armed with two very strong ligatures. A ligature is then tied very firmly on each side, and the isthmus of the thyroid body divided between them. A little dissection with a blunt instrument denudes the trachea to the required extent, and an opening can be made without danger of a drop of blood being drawn into it.

The ligatures which have been thus secured, serve the purpose of fixing the trachea, if desirable, and they may be tied behind the neck so as to raise it forward, and keep the wound open. I never open the trachea until the hemorrhage is stopped, and a large surface of it has been quite denuded.

2. *Keeping the opening in the trachea pervious without resorting to a tube.*—The objections to a tube are twofold: 1st, When the operation is performed for the extraction of a foreign body, it prevents its exit; and it is desirable to leave this opening in such a state that the foreign substance may escape whenever it becomes loosened from its situation in the bronchia. 2d,

* *Gazette des Hopitaux*, for 1854, p. 59.

In tracheotomy for croup, the prolonged sojourn of the tube has been considered, by the most eminent surgeons, as a cause of the pneumonias which so frequently are the cause of death.

The necessity for using the tube I avoid by the following means: Having denuded the trachea, insert a small suture needle, armed with a ligature, beneath two of its rings. Withdraw the needle, and, drawing gently upon the thread, make a semi-circular incision on one side, so as to form a valve, readily opened by drawing upon the thread. The opening thus formed can be kept patent or be allowed to close at will.

This is a matter, perhaps, of much greater consequence than might be supposed without reflection. Most surgeons have found their operations for tracheotomy less successful than they had reason *a priori* to expect, and this has been attributed to the direct entrance of cold air into the lungs. Trousseau and Guersent have both advised that the air inhaled at that time should be quite warm without being too hot.

I have ascertained, by direct experiment, that tracheotomy on dogs is followed by a diminution of temperature. I made a small opening in the skin of a strong dog, upon the side of the thorax, and introducing a thermometer, I found the temperature 98° Fahrenheit. I then performed tracheotomy, and at the end of forty-five minutes the temperature had fallen three degrees. This experiment was repeated twice. In one case the result was the same; in the other, when the temperature before the operation was only 95°, it remained the same after.

Every experienced physiologist knows the facility with which young animals die from reduction of temperature, and when we consider the tender age and depressed state of most of the subjects of tracheotomy, it is reasonable to believe that a reduction such as we have noticed is capable of producing, directly or indirectly, the most injurious consequences. Impressed with this belief, I some years since contrived a cover for the opening into the trachea, composed of wire gauze and sponge, to retain the warmth and moisture of the expired air, and communicate them to that which is inspired. The difficulty of procuring and using this cover, prevented me from publishing an account of it. It afterward appeared to me that the same object might be in a great degree attained by making a valvular opening, so that the air could be directed through the larynx whenever its issue below might not be necessary.

In croup this may be impossible to any great degree, but in the case of foreign bodies, my experience leads me to expect that it will be found both useful and of easy execution.

